# Exploratory Insights on Epidemiology, Genomic Features and Pangenome Analysis of NDM-1-Positive Carbapenem-Resistant *Acinetobacter baumannii* Isolates from Costa Rica

**DOI:** 10.3390/antibiotics15040393

**Published:** 2026-04-12

**Authors:** Jose Arturo Molina-Mora, Daniel Cascante-Serrano, Leana Quirós-Rojas, Gian Carlo González-Carballo, Xavier Araya, Elvira Segura-Retana, Heylin Estrada-Murillo, Stefany Lozada-Alvarado, Mariela Alvarado-Rodríguez, Javier Alfaro-Camacho, Fernando García-Santamaría

**Affiliations:** 1 Centro de Investigación en Enfermedades Tropicales and Facultad de Microbiología, Universidad de Costa Rica, San José 11501-2060, Costa Rica; fernando.garcia@ucr.ac.cr; 2 Caja Costarricense del Seguro Social, San José 10105-1000, Costa Rica; dcascante.serrano@gmail.com (D.C.-S.); lmquirosr@ccss.sa.cr (L.Q.-R.); gcgonzal@ccss.sa.cr (G.C.G.-C.); reivaxares@gmail.com (X.A.); eesegurar@ccss.sa.cr (E.S.-R.); hestradam@ccss.sa.cr (H.E.-M.); wjalfaro@ccss.sa.cr (J.A.-C.); 3 Laboratorio Clínico y Banco de Sangre, Universidad de Costa Rica, San José 11501-2060, Costa Rica; betty.lozada@ucr.ac.cr (S.L.-A.); mariela.alvarado_r@ucr.ac.cr (M.A.-R.)

**Keywords:** carbapenem-resistant *Acinetobacter baumannii* (CRAB), Costa Rica, genomic surveillance, antimicrobial resistance, whole-genome sequencing, epidemiology, resistance rate, Latin America, Prevalence

## Abstract

**Background:** Carbapenem-resistant *Acinetobacter baumannii* (CRAB) is a critical pathogen associated with severe hospital infections and high antimicrobial resistance. Despite the global significance of *A. baumannii*, there are limited data from Costa Rica regarding the resistance rate and genomic characteristics of CRAB. **Methods**: This study aimed to provide initial and exploratory epidemiological data on infections caused by *A. baumannii* and CRAB isolated in Costa Rica and to gain insights on the genome of selected strains, focusing on their resistance determinants and phylogenetic relationships. **Results:** Based on data from five main hospitals in Costa Rica, resistance rate to carbapenems was estimated at 9.8% to imipenem and 6.1% to meropenem. From 190 carbapenem-resistant clinical isolates available in a local collection, seven *A. baumannii* strains were identified, all showing resistance to carbapenems and carrying the *bla*_NDM-1_ gene. Whole-genome sequencing of two strains yielded two distinct MLST profiles (Pasteur scheme: ST-150 for strain IPAT15 and ST-250 for IPAT72), as well as variations in the number and identity of plasmids, genomic islands, and other elements of the mobilome. Both isolates carried ten antimicrobial resistance genes, which are predicted to be harbored in plasmids for IPAT15, unlike the chromosomal determinants in IPAT72. A pangenome analysis of 878 genomes from a public database identified over 51,000 genes, with only 1338 (2.6%) forming the core genome. Phylogenetic analysis and assignation of international clones (ICs) showed predominance of IC2. Isolates from Costa Rica clustered near IC9 and shared some resistance determinants, but they were not directly assigned to an IC. **Conclusions:** Overall, this study provides exploratory insights regarding the occurrence of CRAB in Costa Rica using epidemiological and genomic data, with profiles that are comparable to other regions in Latin America and diverse genomic resistance determinants. While this study does not show the whole landscape of CRAB in Costa Rica, these data constitute an initial approach for improving clinical management and public health responses to CRAB infections, to ultimately improve outcomes for patients affected by this pathogen.

## 1. Introduction

*Acinetobacter baumannii* has emerged as one of the most challenging pathogens in healthcare settings, particularly in intensive care units where it causes significant morbidity and mortality [1]. *A. baumannii* is a critical pathogen causing nosocomial, community-acquired, and opportunistic infections, including bloodstream infections, urinary tract infections, pneumoniae, meningitis, and wound infections, particularly in chronically ill and immunocompromised individuals [2]. This pathogen has garnered increasing attention due to its remarkable ability to develop antimicrobial resistance, including last resource antibiotics such as carbapenems, and persist in hospital environments [3]. Carbapenem-resistant *A. baumannii* (CRAB) is a top-priority pathogen according to the World Health Organization [4,5] and a member of the “ESKAPE” group [6], in which limited treatment options are available with pharmacokinetic restrictions (toxicity) [7]. In a 2025 report, Latin America showed highly variable resistance rates of CRAB ranging from 0% to 90%, highlighting its significant but heterogeneous burden across the region [8].

The resistance mechanisms to several antimicrobials employed by *A. baumannii* are diverse and multilayered [1]. In a recent study, a total of 185 unique antimicrobial resistance genes were identified, with majority of them associated with efflux pump and β-lactamase coding genes, among 609 genomes deposited from diverse geographic regions worldwide [9]. *Acinetobacter* spp. are intrinsically resistant to several commonly used antimicrobials including aminopenicillins, first- and second-generation cephalosporins and chloramphenicol [10]. Resistance to β-lactam antibiotics is primarily mediated by β-lactamases. *A. baumannii* isolates carry a chromosomal, non-inducible AmpC β-lactamase. Extended-spectrum β-lactamases, such as TEM, SHV, and CTX-M types, have also been described in clinical isolates. Carbapenem resistance in *A. baumannii* is mediated by Amber class A *Klebsiella pneumoniae* carbapenemases (*KPC*), Amber class B metallo-β-lactamases, including NDM, VIM, and IMP, and Ambler class D β-lactamases, such as OXA-type carbapenemases [3,11]. In a global cohort study, 96% of *A. baumannii* isolates were reported to harbor acquired carbapenemase genes [12]. Moreover, OmpA is the most abundant outer-membrane protein in *A. baumannii* and it has been considered as the mechanism involved in the resistance to imipenem in several outbreaks. Additionally, membrane modifications and active efflux systems, notably the AdeABC efflux pump system, contribute to its formidable resistance profile [2]. These and other mechanisms, often working in concert, have led to the emergence of carbapenem-resistant strains, which are associated with mortality rates ranging from 35% to 60% [3]. On the other hand, genomic surveillance studies have revealed the complex mechanisms underlying the adaptability and pathogenicity of CRAB strains [13]. The success of the pathogen in hospital settings is attributed to its extraordinary genomic plasticity, which enables rapid adaptation to environmental stresses and antimicrobial pressures [14]. This adaptability is further enhanced by its capacity to form biofilms and utilize sophisticated quorum sensing systems for bacterial communication [2], as well as trigger central molecular responses to stressors, as in other species [15,16].

The successful management of *A. baumannii* and CRAB infections requires a coordinated approach combining enhanced surveillance systems, stringent infection control measures, and the continued development of therapeutic strategies [13]. In this sense, genomic surveillance of CRAB and other carbapenem-resistant bacteria is essential for public health and clinical practice [17]. It helps monitor the emergence of new resistant variants and their spread within hospitals and communities, distinguishing whether cases belong to the same epidemic clone or different strains, which facilitates targeted control measures such as patient isolation and environmental disinfection [18]. Genomic analysis also identifies the genes and mechanisms of resistance, including carbapenemases and other determinants carried on chromosomes, plasmids or transposons [11,18]. Additionally, it allows the tracking of high-risk lineages and international clones (ICs) through genomic typing methods, including Multi-Locus Sequence Typing (MLST) [19]. Thus, understanding the genomic basis of its resistance and virulence helps in developing effective interventions and preventing its spread in healthcare settings [2,11]. As reported in the PubMLST database [20], two MLST schemes are available for *A. baumannii*. Both are based on seven housekeeping genes but offer different advantages [8]. The first, described by Bartual et al. in 2005 [21], is commonly referred to as the “Oxford” scheme, since it was originally hosted on the PubMLST site at the University of Oxford. It provides a fine discriminatory resolution but is affected by recombination events, making it particularly useful for outbreak analysis but less reliable for evolutionary studies [8]. The second, described by Diancourt et al. in 2010 [22], is known as the “Pasteur” scheme, to distinguish it from the Oxford scheme. This scheme, although providing slightly lower resolution, is more stable and therefore better suited for studying long-term evolutionary trends and population structure [8].

Genomic data from Latin America indicate a distinct epidemiological landscape compared to IC2-predominance in the Global North, with greater prominence of clones such as IC4 and IC5, alongside emerging and underrepresented lineages that remain poorly captured in global databases [8]. Despite the global recognition of genomic surveillance for antimicrobial-resistant bacteria, Latin America faces significant gaps compared to other regions [23,24]. These include underrepresentation in genomic databases, insufficient infrastructure and resources, shortages of trained personnel, and lack of real-time data generation and analysis [23,25]. As a result, sequencing capacity in the region remains low, even during periods of increased investment such as the COVID-19 pandemic [26,27,28]. Also, strengthening regional collaboration and participation in global networks is essential to improve genomic surveillance, support evidence-based decision-making, and ensure more equitable access to the benefits derived from pathogen genomic data for CRAB, as well as resistant bacteria in general [24,29].

For example, in Costa Rica, reports on cases, prevalence and resistance rates of CRAB remain very limited. In many publications, frequency and prevalence are not reported for this country due to the lack of data [13,30,31,32], and a similar situation applies to genomic information, where only a single study described specific profiles in four strains (outbreak caused by an ST126 strain harboring *bla*_NDM-1_ and *bla*_OXA-58_ genes) [33], but other data were not available [34]. At the local epidemiological surveillance level, prevalence and resistance rates are assumed as relatively low and comparable to those observed in several Latin American countries [13,30,31,32].

Given the global significance of *A. baumannii* and the limited data from Costa Rica regarding the resistance rate and genomic characteristics of CRAB, this study aims to provide initial and exploratory epidemiological data on infections caused by *A. baumannii* and CRAB isolated in Costa Rica and to gain insights on the genome of selected strains focusing on their resistance determinants and phylogenetic relationships, as a model to strengthen genomic surveillance and guide decision-making against antimicrobial resistance.

## 2. Results

In this study, cases of CRAB isolated from clinical centers in Costa Rica were evaluated. According to surveillance data (Table 1), national hospitals (all located in the main metropolitan area, covering 90% of Costa Rican population) reported between 50 and 98 isolates per year (2023–2024). Carbapenem resistance (imipenem and meropenem) remained between 1% and 10% in two hospitals, while in a third center, imipenem resistance reached 23.5% in 2024, although meropenem resistance remained low (3%). In regional hospitals (outside the main metropolitan area), the number of isolates was very limited (one to six per year), and nearly all remained susceptible, except for one case of meropenem resistance in 2023.

From a local collection of 190 clinical carbapenem-resistant isolates, only seven *A. baumannii* strains were identified, all with resistance to imipenem and meropenem (Table 2). Molecular testing confirmed the presence of the *bla*_NDM-1_ gene in all cases, persistently detected at least since 2017, when the first isolate in the collection was documented.

Whole-genome sequencing of two isolates (IPAT15 and IPAT72) yielded high-quality assemblies that met the minimum standards of the 3C criterion (Table 3). The assembled genomes showed numbers of contigs and N50 values consistent with the sequencing strategy. Both isolates exhibited 100% completeness and similar GC contents of approximately 39%. Regarding structural genome annotation, IPAT72 presented a slightly higher number of predicted genes (3974 vs. 3910) and coding sequences (3906 vs. 3846).

Functional annotation revealed distinct MLST profiles in the Pasteur and Oxford schemes: ST-150/ST-3294 for IPAT15 and ST-250/ST-1739 for IPAT72. Variations were also observed in the number and identity of plasmids, genomic islands, and insertion sequences. With respect to prophages, seven were identified in IPAT15 (one complete) and eight in IPAT72 (three complete). For CRISPR-Cas systems, IPAT15 harbored a complete CAS-TypeIF system, whereas only remnants were detected in IPAT72.

Regarding antimicrobial efflux pumps (resistance–nodulation–cell division, small multidrug resistance, or major facilitator superfamily), at least 12 were identified in IPAT15 and nine in IPAT72. Excluding gene coding for efflux pump systems, both isolates carried ten antimicrobial resistance genes, though with different profiles (Table 3). For IPAT15, *bla*_ADC-163_ and *bla*_OXA-121_ were found in the chromosome, while *bla*_NDM-1_ and *bla_CARB-_*_16_ were found in the plasmid MK134375. In IPAT72, antimicrobial resistance genes were only found in chromosomal sequences, including *bla*_ADC-216_, *bla*_CARB-14_, *bla*_NDM-1_, and *bla*_OXA-407_. More details about antimicrobial resistance genes by sequence are available in the Supplementary Material Table S2.

In general, these findings regarding genome annotation contrasted with the reference strain (GCA_009035845.1), which contains 3683 CDS, two plasmids, 39.0% GC, six prophages (two complete), four insertion sequences, and only three resistance genes (*sul2*, *bla*_OXA-98_, and *bla*_ADC-25_).

To further explore the particularities of the two sequenced isolates in a global context, sequenced genomes were compared with 876 publicly available sequences, for a total of 878 sequences. Pangenome analysis identified more than 51,000 genes, of which 1338 (2.6%) belonged to the core genome, while 46,370 (90.6%) were present in fewer than 15% of the strains (Table 4).

**Figure 1:**
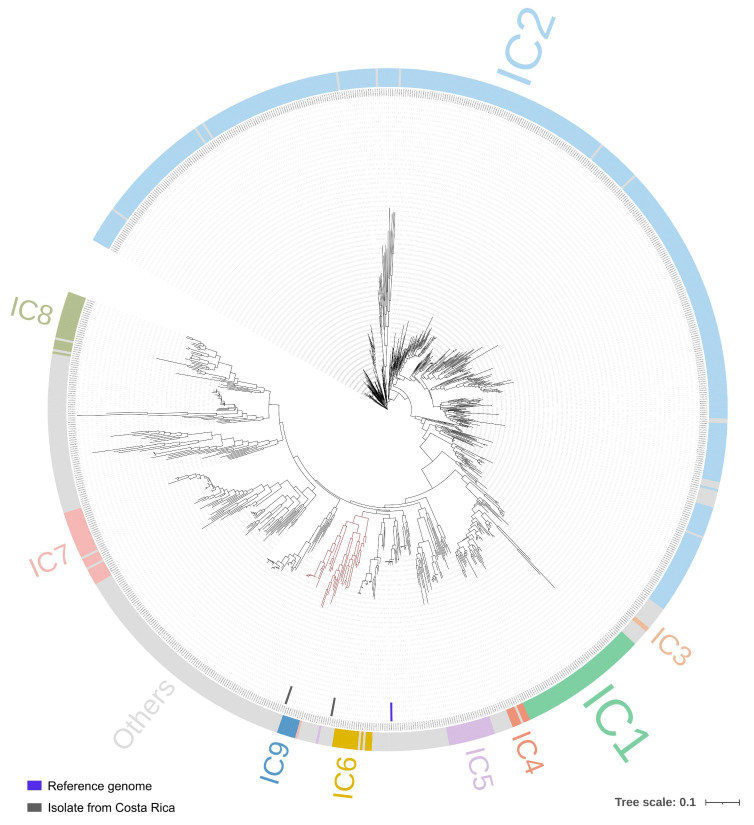
Comparative genomic analysis of 878 *A. baumanni* strains using a pangenome strategy. Pangenome analysis and genome annotation showed that overall gene content separates and clusters genomes according to their international clone (IC#) profile, represented by colors; gray indicates genomes not assigned to any international clone. In the tree, a specific group with branches highlighted in red corresponds to the isolates sequenced (with internal black strips) in this study; this branch is enlarged in Figure 2.

Based on this pangenome information, a phylogenetic tree was constructed (Figure 1), showing a distribution consistent with international clones (ICs), with IC2 predominating and other groups occurring at lower frequencies. The two cases isolated in Costa Rica were not directly assigned to an IC but clustered near IC9, forming part of a specific cluster.

**Figure 2:**
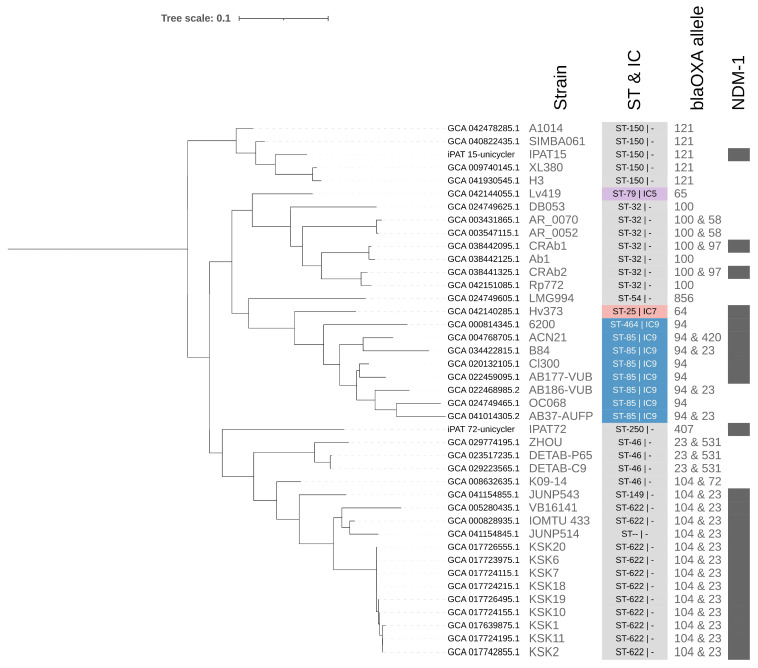
Comparative genomic analysis based on the pangenome approach for the clade harboring the CRAB isolates IPAT15 and IPAT72. Subtree derived from the pangenome analysis (red branch in the tree, Figure 1), including the isolates sequenced in this study. A close relationship with genomes belonging to the international clone IC9 is observed; however, the Costa Rican cases (IPAT15 and IPAT72) do not cluster within the same clade, indicating distinct genomic profiles. Both harbor the *bla*_NDM-1_ gene but differ in other markers, such as *bla*_OXA_ alleles, as well as in overall gene content, explaining their separation in the tree. MLST assignment, *bla*_OXA_ alleles, and *bla*_NDM-1_ presence are indicated. Gray denotes genomes not assigned to an international clone, and a dash (–) indicates undefined ST. Coloring follows the IC scheme in Figure 1, with IC9 predominating in this branch and additional representation of IC5 and IC7.

Detailed analysis of this clade (Figure 2) showed that a large proportion of genomes carried the *bla*_NDM-1_ gene. An association between subclades and specific *bla*_OXA_ alleles was also observed. Interestingly, the cases isolated in Costa Rica not only exhibited distinct genomic features but also clustered separately, consistent with their different MLST and mobilome profiles. This indicates possible independent evolutionary histories rather than recent clonal transmission between the two cases, and they are not related to an outbreak. However, due to limited data, these results do not show the whole landscape of CRAB in Costa Rica.

**Figure 3:**
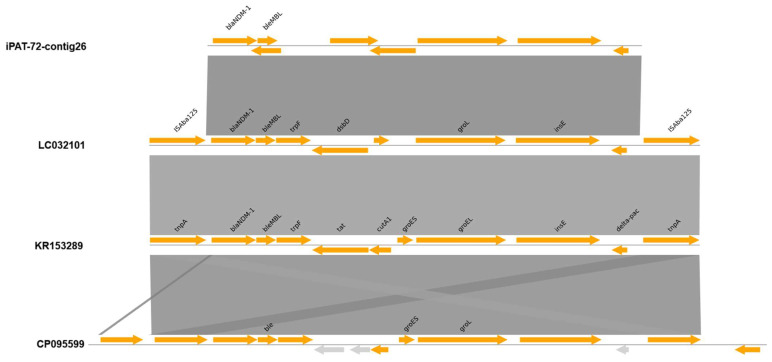
Genomic context associated with the presence of the *bla*_NDM-1_ gene in a CRAB isolated in Costa Rica (IPAT72). The genomic fragment containing the *bla*_NDM-1_ gene in the IPAT72 genome was analyzed by comparison using BLAST, selecting sequences with full coverage and identity. Three records were selected, showing slight annotation differences attributable to the tools used during database submission. Overall, the architecture of this genomic region includes multiple antimicrobial resistance determinants, not only *bla*_NDM-1_ but also other associated genes (e.g., *ble*_MBL_ and *groES*), and is flanked by insertion sequences associated with potential genetic mobility.

Finally, the genomic context associated with the presence of the *bla*_NDM-1_ gene in the study strains was analyzed using comparative genomics (Figure 3). Structural annotation of the complete contig #26 from strain IPAT72 revealed a region identical to that observed in several genomes, in both plasmids and chromosomes. Despite 100% identity, differences in the genome annotation are evident (gene predictions are different depending on the sequence). The three cases with identical sequences are (GeneBank ID) LC032101, KR153289, and CP095599; however, in these genomes, the region extends across larger genomic segments. Comparative analysis showed the presence of *bla*_NDM-1_ as well as other resistance determinants, such as *ble*_MBL_, *groEL* and others; the region of the IPAT72 contig is flanked by the same insertion sequence.

## 3. Discussion

Current epidemiological data indicates a concerning trend in the global prevalence of *A. baumannii*, particularly in its multidrug-resistant lineages such as CRAB [14]. The economic impact on healthcare systems is substantial, encompassing extended hospital stays, increased treatment costs, and the necessity for enhanced infection control measures [3]. The spread of CRAB has become a significant public health concern, prompting the World Health Organization to classify it as a critical priority pathogen requiring urgent attention [2].

Given the global significance of *A. baumannii* and its increasing resistance patterns, understanding of its molecular epidemiology in specific geographical regions becomes crucial for effective control measures [35]. This need is particularly critical for Latin America, which has some of the highest CRAB burdens globally [12]. However, until now, the current prevalence or resistance rate of CRAB in Costa Rica has been referred to as unknown in several global studies [13,30,31,32]. Data from 2002 inferred 3% of CRAB prevalence in this country [13], but no recent information is available. In this study, resistance rate data on CRAB infections, whole-genome sequencing and pangenome analyses were used to provide exploratory insights into the resistance determinants and phylogenetic relationships of selected *A. baumannii* strains isolated in Costa Rica. Although the genomes analyzed are not fully representative of all CRAB cases in the country, limiting broader interpretation, these findings constitute an important initial step toward strengthening genomic surveillance of antimicrobial resistance in Costa Rica. This work highlights the potential of integrating genomic approaches in resource-limited settings such as Latin America, while underscoring the need for expanded and sustained efforts in the region.

In a first analysis, based on the data collected from this study, it was shown that the reported number of CRAB isolates in Costa Rica remains relatively low in comparison to other pathogens, and current resistance rate was estimated at 9.8% to imipenem and 6.1% to meropenem. These results are in line with previous studies in the region, in which other Central American countries have reported the lowest values of CRAB resistance rate in Latin America, unlike other places such as Argentina and Ecuador reaching >80% [13].

However, unlike other centers, Hospital #1 showed a notable increase in cases from 2023 to 2024 (1.1% to 23.5%), particularly in imipenem resistance. Importantly, no significant changes in molecular diagnostic strategies or tools were reported between these years in this center, suggesting that the increase is unlikely due to methodological differences. This rise coincides with a reported outbreak driven mainly by Enterobacteriaceae carrying the *bla*_NDM-1_ gene, with sequencing data suggesting mobilization via a megaplasmid. Therefore, it cannot be ruled out that the increase in carbapenem resistance in *Acinetobacter* in 2024 at this hospital is linked to this outbreak [36].

The resistance rate of CRAB varies widely between countries due to a combination of healthcare, epidemiological, and surveillance-related factors; in some settings such as Costa Rica, its relatively low frequency is notable given that in similar scenarios in other Latin American countries it represents a major problem, while *Pseudomonas aeruginosa* appears to be more prevalent and clinically significant, even with high levels of carbapenem resistance—an observation that could suggest potential niche-related dynamics, but it currently lacks supporting evidence and remains an open question highlighted in this study.

In addition, the reported resistance rate values in our study should be interpreted as exploratory estimates. Although the information provided by hospitals potentially covered 90% of Costa Rica’s population, the analysis was performed using reported cases and not a systematic or controlled prevalence nor resistance rate study. Therefore, these values are influenced by factors such as laboratory access, sampling, and testing workflows (number of infections, laboratory submissions, culture rates, and antimicrobial susceptibility testing). In the context of limited national data, these findings provide an important initial reference point for CRAB in Costa Rica. At the same time, they highlight the need for more comprehensive and systematic studies that integrate clinical, microbiological, and epidemiological data to generate more accurate resistance rate estimates [37].

On the other hand, CRAB genomic surveillance supports clinical decision-making by complementing phenotypic resistance data, which guides treatment choices for complex infections. However, Latin America faces significant challenges in the genomic surveillance of antimicrobial-resistant bacteria, including underrepresentation in genomic databases, limited infrastructure and resources, and shortages of trained personnel, which hinder real-time data generation and analysis [23,24,25]. These gaps have practical consequences. As we reported in a previous study, an automated diagnosis system (e.g., PCR-based platforms) failed to detect locally prevalent variants of the IMP gene, which is relevant in Latin America but not in the Global North, leading to inaccurate results for patients and clinicians [38].

While initiatives such as Pathogen Access and Benefit Sharing (PABS) have promoted global progress [29], including the development of regional data-sharing nodes led by countries like Costa Rica and Mexico (https://www.pathogensportal.org/pathogens-portal-nodes), these efforts remain insufficient. Strengthening regional collaboration and participation in global networks is essential to improve surveillance, optimize diagnostic tools for local contexts, and ensure equitable access to the benefits derived from genomic data [23,25,29].

Regarding genomic analysis for CRAB in Costa Rica, a single study had conducted whole-genome sequencing with isolates associated with an outbreak of CRAB between October 2020 and April 2021, resulting in three fatalities among four infected patients. The outbreak was caused by an ST-126 strain, a sequence type not previously reported in Latin America, which exhibited a concerning multidrug-resistant profile. Genomic analysis revealed the presence of a 72 kb plasmid carrying both *bla*_NDM-1_ and *bla*_OXA-58_ genes [33]. ST-126 is embedded within IC2, the most globally prevalent epidemic lineage and one of the principal clones in Latin America, with characteristic β-lactamase repertoires (*bla*_OXA-66_, *bla*_ADC-30_, *bla*_OXA-72_) [39,40].

Now, in this study we are reporting the genome sequence of two CRAB isolates with different genomic features in Costa Rica, indicated with an MLST (Pasteur schema) profile ST-150 (strain IPAT15) and ST-250 (IPAT72). None of the genome sequences were directly assigned to an IC (under the current schemas), although clustered near IC9.

ICs of *A. baumannii* are major drivers of hospital-acquired infections and outbreaks, and are widely distributed across multiple countries [8,35]. Clinically, these high-risk ICs are strongly associated with multidrug resistance, including resistance to carbapenems mediated by OXA-type and other carbapenemases [41]. This profile as IC limits treatment options, often leaving only last-resort drugs such as colistin, and is associated with high morbidity and mortality, especially in critically ill patients [42].

However, assigning an isolate to an IC (based on MLST profile) is not always straightforward; it depends heavily on the availability and quality of genomic and epidemiological data [19]. Thus, from an epidemiology perspective, these clones spread efficiently across hospitals and countries, facilitated by patient movement and environmental persistence [19]. Yet, in regions like Latin America, and particularly Costa Rica, there is still limited genomic surveillance data, making it difficult to determine whether circulating strains truly belong to established IC, even for the cases included in this study (close to IC-9). With improved data representation, it may become clearer whether local isolates are part of these global lineages or represent distinct regional variants. Notably, many strains in these regions already harbor carbapenem resistance genes and OXA variants that have been previously linked to globally disseminated, high-risk clones, suggesting that potential connections remain to be fully characterized [19,22].

A recent study, conducted in a tertiary hospital in Paraguay, showed that 98% of the strains were resistant to carbapenems and their genomic epidemiology analysis included 200 genomes that were found in the main international clones IC1, IC2, IC4, IC5 and IC7 [43]. Also, a global survey of 313 CRAB from 114 hospitals in 47 countries found that 92% of isolates corresponded to international clones IC1–IC8, with IC2 predominating worldwide (63%). Acquired OXA-type carbapenemases were detected in 96% of isolates, mainly OXA-23-like and OXA-40-like, while metallo-β-lactamases were rare (~2%) [12]. These data explain the persistently high rates of carbapenem resistance across regions and the clonal nature of hospital endemicity and outbreaks. IC5 (44/313; 14%) was largely confined to Latin America, contrasting with the IC2 predominance elsewhere, and carried a distinct resistance-gene profile, highlighting region-specific epidemiology and the need for tailored infection prevention strategies [12].

Regarding other ICs for *A. baumannii*, the IC1 is a long-standing lineage with repeated involvement in hospital outbreaks and carbapenem resistance via OXA-type carbapenemases, often OXA-23, across Europe, the Middle East and parts of Asia [39,40,44], but not for Latin America [45]. While the latest global super-lineage analysis prioritizes IC2 growth [40], it supports the long-standing view that multiple successful clades co-circulate and can dominate regionally [13,30].

In addition, a strong correlation has been reported between IC profiles and variants of the *bla*_OXA-51_ gene, commonly referred to as *bla*_OXA-51-like_ alleles [46]. This finding was evidenced in our analyses, where consistent patterns of occurrence of specific *bla*_OXA_ alleles were observed for the same MLST profiles, according to the pangenome-based phylogenetic analysis. For instance, in IC9, *bla*_OXA-94_ was associated with ST-85 and ST-464, while in the cluster containing IPAT15, *bla*_OXA-121_ was linked to ST-150. In the case of the IC9, this association is in line with the definition presented in a recent study [19].

With respect to the mobilome, the studied genomes had specific genomic features including different plasmids, insertion sequences, and phages. Again, this is in line with the strong capacity of CRAB to acquire foreign genetic material. Plasmids such as MK134375 in CRAB (strain IPAT15) have been previously reported [47], as well as CP021785 and CP021783 (found in IPAT72), in this bacterial group and other species [48,49]. MK134375 and CP021783 are small rep-3 plasmids that are often cryptic [50,51,52]. CP021785 is self-transmissible and usually reported as rich in resistance determinants and mobile genetic elements [50,53,54,55]. Despite these findings, the short-read sequencing approach used in this study was not sufficient to fully resolve plasmid structures, which represents a limitation. In many cases, short-read data alone cannot accurately reconstruct complete plasmids due to repetitive regions and structural complexity [56]. This limitation could be addressed by incorporating long-read sequencing strategies, which in numerous instances enable the complete resolution of plasmid sequences [57]. Such approaches would provide more comprehensive information not only on the genes and mobile elements carried by these plasmids and their identities, but also on their structural organization [57,58]. In addition, improved resolution would facilitate more robust analyses of plasmid dissemination patterns and potential transmission pathways, including the ability to investigate events involving plasmids shared across bacterial species [56,57].

Antimicrobial genes detected in the strains, such as *bla*_ADC-163/216_, *bla*_CARB-16/14_, *bla*_NDM-1_, and *bla*_OXA-121/407_, confer resistance to several groups of antimicrobials such as aminoglycosides, *β*-lactams and carbapenems. In the case of *bla*_NDM-1_, *A. baumannii* isolates hosting these enzyme genes are highly observed over the world, particularly in eastern countries and this gene was found on several plasmids [59]. In our study, *bla*_NDM-1_ and *bla_CARB-_*_16_ were predicted to be found in the plasmid MK134375 for strain IPAT15, although IPAT72 harbors *bla*_NDM-1_ in the chromosome and not in plasmids.

The genomic context associated with the presence of the *bla*_NDM-1_ gene was study for IPAT72, in which identical regions of the assembled fragment have been observed in several genomes, in both plasmids and chromosomes. *bla*_NDM-1_ is accompanied by *ble*_MBL_, *groEL* and other elements, and the contig is flanked by two identical insertion sequences. This suggests the participation of mobile elements in the spread of these resistance determinants. However, the whole context was not solved. The resolution for short-read sequencing resulted in multiple fragments or contigs, without a fully reconstructed circular sequence nor definitively resolved architecture of the genomic region, which is in line with the feature of this type of technology [60,61,62,63]. In the IPAT15 strain, the contig containing the *bla*_NDM-1_ gene was extremely short, preventing determination of its genomic context within the plasmid. This limitation also created challenges during structural annotation. Again, these results highlight the need to employ long-read sequencing technologies to clarify the nature of these elements and their potential relationship to plasmids previously identified in Costa Rica associated with outbreaks [33,36].

As previously reported for CRAB, *bla*_NDM_ variants exhibit the greatest diversity and are found in several genomic contexts [64], which are consistent with our observations, once again indicating the ability of *Acinetobacter* species to adapt and exchange genetic determinants through multiple mechanisms [22,65].

Regarding the pangenome, 51,174 genes were found within 878 strains, with a core genome (>95% strains) composed of >1300 genes. Considering similar approaches, contrasting results and purposes have been found in other studies. A study in 2019 revealed that the core genome and pangenome were defined with 2221 and 19,272 genes, respectively [65]. In another study, authors employed a comprehensive pangenome analysis to investigate a group of multidrug-resistant *A. baumannii* strains, in which two clusters were identified based on encoding β-lactamases and multidrug efflux pump as potential targets for drug design [66]. A similar approach was used by Hassan et al. using a pangenome analysis to determine potential core vaccine targets using a pangenome analysis [67].

In addition, the observation that over 90% of the accessory pangenome genes are present in fewer than 15% of strains highlights the remarkable versatility of this organism in sharing and transferring genetic material. Each strain, therefore, harbors a distinct gene composition that confers high diversity, which is reflected not only in genomic profiles (such as MLST) but also in potential traits linked to environmental adaptation, virulence, and antimicrobial resistance [66,67]. These general patterns, identified in the analysis of 878 genomes, are also evident at the individual genome level in isolates sequenced in this study.

Jointly, the findings of this study underscore the importance of investigating CRAB and other carbapenem-resistant bacteria, as the diversity and characteristics of isolates are highly context-specific [35,38]. Expanding epidemiological monitoring and making data on cases, resistance rate, phenotypes, and genomic sequences publicly available are essential steps to maximize the benefits for clinical management, decision-making, diagnostic development, and therapeutic strategies [68], while also accounting for strains circulating in different geographic regions. Such efforts strengthen integration into regional and global networks and ultimately improve outcomes for patients affected by infections caused by this pathogen [8,35].

Finally, this study has several limitations: resistance rate was inferred exclusively from cases reported in selected hospitals from Costa Rica, without considering the different levels of complexity throughout the country’s health system or systematic assessment to infer it. The selection of isolates for molecular analysis by PCR and whole-genome sequencing was based on availability and therefore does not fully represent the actual circulation of this pathogen. It is necessary to precisely define the genetic context and mobile elements harboring resistance genes through long-read sequencing and complementary approaches. In addition, the analyses did not include other relevant contexts, such as animal or environmental reservoirs, which are critical to obtaining a better understanding and expanding the local dynamics of CRAB.

## 4. Materials and Methods

### 4.1. Data Source

The CRAB strains are part of the collection of 190 carbapenem-resistant bacterial isolates from clinical origin that is maintained at the University of Costa Rica, Costa Rica. Isolates corresponded to infection cases in humans, and each CRAB isolate was obtained from a different individual (i.e., no multiple isolates from the same person). The epidemiological data on infection cases were obtained from general statistics in consensus reports for 2024 and 2025 in health centers. The hospitals correspond to the three main public hospitals in the country (classified as “Class A”), which cover approximately 90% of the population based on the number of registered individuals (4,641,981 out of a total population of 5,170,030 in Costa Rica). Additionally, two regional hospitals located outside the metropolitan area were included. Patient information was not available due to limitations established with the approval and the nature of the project (epidemiological study).

### 4.2. Identification and Antimicrobial Susceptibility Testing

Species-level identification and antimicrobial susceptibility testing were performed using the VITEK^®^ 2 automated system (bioMérieux, Marcy-l’Étoile, France), following the manufacturer’s instructions with cards for Gram-negative bacilli. The minimum inhibitory concentrations (MICs) were determined by the system, and results were interpreted according to the Clinical and Laboratory Standards Institute guidelines CLSI M100 (35th Ed.).

### 4.3. Genomic DNA Extraction and Quantification

For genomic DNA extraction, NucleoSpin^®^ Tissue kit (Macherey-Nagel, Düren, Germany) was used according to the manufacturer’s instructions for bacteria. DNA purity was evaluated using a NanoDrop™ 2000 (Thermo Fisher Scientific, Waltham, MA, USA) and DNA concentration was quantified with a Qubit™ 4 Fluorometer (Thermo Fisher Scientific, USA).

### 4.4. Automated PCR Analysis for Carbapenemase Gene Detection

The GeneXpert^®^ system (Cepheid, USA) with the Xpert^®^ Carba-R assay (qPCR), following the manufacturer’s instructions, was implemented for the identification of carbapenemase genes, including *bla*_KPC_*, bla*_NDM_*, bla*_VIM_*, bla*_OXA-48_*,* and *bla*_IMP_. Results were interpreted automatically by the system’s software within the GeneXpert.

### 4.5. Whole-Genome Sequencing, Assembly and Annotation

Only two CRAB isolates were available for whole-genome sequencing; DNA was extracted as previously indicated. Genomic DNA was prepared using a standard Illumina DNA shotgun library preparation protocol with a subsequent analysis by sequencing with a NovaSeq X Plus device (Illumina Inc., San Diego, CA, USA) at Novogene, Sacramento CA, USA. More than 1.00 Gb raw data per sample was approximately generated (150 bp, paired end sequencing).

The HPC-UCR computational cluster (Universidad de Costa Rica, https://hpc.ucr.ac.cr/) was used to perform bioinformatic analyses. FastQC v0.12.1 [69] was used to assess reads before and after trimming. Reads were trimmed (including adapter removal and Q > 30) using Trimmomatic v0.38 [70]. Megahit v1.2.9 [71] and Unicycler v0.5.1 [72] were implemented for de novo genome assembly (contigs with length < 1000 bp were discarded), with a final selection of the best assembly based on the 3C criterion, including the number or contigs, genome size, coding sequences, completeness score, and fidelity metrics [60,73].

Structural annotation was performed with Prokka v1.14.6 [74]. Genome functional annotation was achieved using Abricate v1.0.1 (https://github.com/tseemann/abricate) to identify antimicrobial resistance genes, while MLST profile (Pasteur and Oxford schemes) was defined using PathogenWatch v23.5.0 (https://pathogen.watch/). The mobilome was studied using PHASTER v3.0 [75], IslandViewer 4.0 [76], ISfinder v2.0 (https://isfinder.biotoul.fr/) and CRISPRCasFinder 1.1.2 (https://crisprcas.i2bc.paris-saclay.fr/CrisprCasFinder/Index). Plasmids were identified using Mob-suite 3.1.9 [77].

### 4.6. Pangenome and Comparative Analysis

All complete whole-genome sequences of *A. baumannii* were retrieved from NCBI (https://www.ncbi.nlm.nih.gov/datasets/genome/, accessed on 20 April 2025). A total of 876 sequences were downloaded and annotated by MLST using PathogenWatch v23.5.0 (https://pathogen.watch/). See Supplementary Material Table S1 for details. Structural annotation was performed with Prokka v1.14.6 [74], and the resulting annotated files were used to run Roary v3.12.0 [78] for the pangenome analysis. The phylogenetic tree was visualized using Interactive Tree Of Life Tool (iTOL, https://itol.embl.de/) v7 [79]. Based on the MLST profile, each sequence type (ST) was assigned to its corresponding international clone according to [19], and this classification was represented in the figure using different colors; gray indicates genomes that do not belong to any international clone.

In addition, based on the results, the genomic context of the *bla*_NDM-1_ gene found in the local strains was analyzed by comparative genomics. The assembled contig carrying *bla*_NDM-1_ was queried against public databases using BLAST v2.17.0 (https://blast.ncbi.nlm.nih.gov/Blast.cgi), and closely related sequences were selected based on both sequence identity and coverage. Comparisons by alignments using regions from complete genomes (GenBank files) were performed to better resolve the genetic environment and to study flanking regions. Genomic neighborhoods were visualized using the PyGenomeViz WebApp (https://pygenomeviz.streamlit.app/).

## 5. Conclusions

This study provides initial insights into the epidemiology and genomic diversity of CRAB in Costa Rica, within the broader context of Latin America. Epidemiological data from five major hospitals (potentially covering >90% Costa Rican population) revealed resistance rates of 9.8% to imipenem and 6.1% to meropenem, consistent with regional reports, where CRAB burden remains highly variable. The identification of NDM-1-producing *A. baumannii* strains confirms the circulation of highly resistant clones in the country.

Although based on a limited number of isolates, this study highlights relevant findings and serves as a valuable contribution toward building local and regional networking efforts. Whole-genome sequencing of representative isolates revealed demonstrated distinct sequence types (ST-150 and ST-250, Pasteur scheme) and substantial genomic variability, particularly in plasmids, genomic islands, and other mobile genetic elements, underscoring the adaptability and capacity for horizontal gene transfer in *A. baumannii*. Differences in the genomic context of resistance genes—including plasmid-associated *blaNDM*-1—and distinct clustering profiles based on the pangenome further illustrate the dynamic nature of resistance dissemination, including enriched groups such as the IC9 and the presence of locally adapted lineages with distinct resistance determinants.

These findings reflect the distinct and still underrepresented genomic landscape of CRAB in Latin America and demonstrate the potential for integrating genomic approaches into routine surveillance. At the same time, they emphasize that significant gaps remain, including the need for larger datasets, sustained infrastructure, and stronger regional collaboration. Expanding coordinated genomic surveillance, improving data sharing, and fostering networking initiatives will be essential to advance toward more robust, real-time, and equitable public health responses in the region.

## Figures and Tables

**Table 1:** Resistance rates of *A. baumannii* isolates with resistance to carbapenems from five hospitals of Costa Rica, 2023–2024.

Hospital * (Registered Population)	Period	*A. baumannii* Isolates		
		Total	Imipenem-Resistant	Meropenem-Resistant
National #1 1,165,500	2023	88	1 (1.1%)	3 (3.4%)
	2024	98	23 (23.5%)	3 (3.1%)
National #2 1,890,381	2023	52	5 (9.6%)	5 (9.6%)
	2024	50	4 (8.0%)	4 (8.0%)
National #3 1,586,100	2023	62	4 (6.5%)	4 (6.5%)
	2024	65	5 (7.7%)	5 (7.7%)
Regional #1 >330,000	2023	6	0 (0.0%)	0 (0.0%)
	2024	1	0 (0.0%)	0 (0.0%)
Regional #2 >160,000	2023	4	0 (0.0%)	1 (25.0%)
	2024	2	0 (0.0%)	1 (0.0%)
Total cases	2023–2024	428	42 (9.8%)	26 (6.1%)

* More than 90% of the Costa Rican population is registered in one out of the three national hospitals, which are the only three “class A” centers in this country.

**Table 2:** Profile of selected carbapenem-resistant *A. baumannii* isolates from Costa Rica.

Strain	Automated PCR	Date	Imipenem (μg/mL)	Meropenem (μg/mL)	Hospital
IPAT15	*bla*_NDM_	14 October 2023	≥16 (R)	8 (R)	National #1
IPAT72	*bla*_NDM_	14 July 2022	≥16 (R)	8 (R)	National #2
IPAT126	*bla*_NDM_	3 April 2018	≥16 (R)	8 (R)	Private
IPAT127	*bla*_NDM_	26 January 2018	>32 (R)	>32 (R)	Private
IPAT128	*bla*_NDM_	1 July 2017	≥16 (R)	8 (R)	Private
IPAT129	*bla*_NDM_	23 January 2019	>8 (R)	>8 (R)	Private
IPAT130	*bla*_NDM_	11 February 2025	>8 (R)	>32 (R)	Private
IPAT155	*bla*_NDM_	2 September 2025	≥16 (R)	>8 (R)	National #2

**Table 3:** Genome statistics; structural and functional annotation of the genome sequences of two *A. baumannii* isolated in Costa Rica.

Annotations	Strain IPAT15	Strain IPAT72
Contigs	37	54
Total length	4,133,968	4,096,902
N50	444,184	232,944
Completeness (score)	100%	100%
%GC	39.08%	38.84%
Genes	3910	3974
Coding sequences (CDSs)	3846	3906
MLST (Pasteur schema)	ST-150	ST-250
MLST (Oxford schema)	ST-3294	ST-1739
Plasmids	2 CP034093 and MK134375	3 KT346360, CP021785 and CP021783
Genomic islands (IslandPath-DIMOB)	5	8
Insertion sequences	4	3
Phages	7 (one complete)	8 (three complete)
CRISPR-Cas	One complete system: CAS-TypeIF (five genes) and 2 CRISPR (13 and 82 spacers)	Only CRISPR remnants (max two spacers), no Cas proteins
Antimicrobial resistance genes	10 *aac(3)-IId* *, *ant(3*′′*)-IIa, bla*_ADC-163_, *bla*_CARB-16_ *, *bla*_NDM-1_ *, *bla*_OXA-121_, *floR* *, *mph(E)* *, *msr(E)* *, *sul2* *	10 *aac(6*′*)-Ian*, *ant(2*′′*)-Ia*, *ant(3*′′*)-IIa*, *aph(3*′*)-Ia*, *bla*_ADC-216_, *bla*_CARB-14_, *bla*_NDM-1_, *bla*_OXA-407_, *ble*_MBL_, *sul2*

* Found in plasmids.

**Table 4:** Statistics of the pangenome analysis with 878 *A. baumanni* strains.

Genome	Level (Frequency)	Number of Genes
Core genome	Strict core genes (99% ≤ strains ≤ 100%)	528
	Soft core genes (95% ≤ strains < 99%)	810
Accessory genome	Shell genes (15% ≤ strains < 95%)	3466
	Cloud genes (0% ≤ strains < 15%)	46,370
Total	(0% ≤ strains ≤ 100%)	51,174

## Data Availability

Genome sequences can be found in the European Nucleotide Archive (ENA) Repository, accession PRJEB96900 (https://www.ebi.ac.uk/ena/browser/view/PRJEB96900); other processed data supporting the findings of this study are available within the paper and its Supplementary Information. Raw sequencing data are available from the corresponding author upon reasonable request.

## Supplementary Materials

The following supporting information can be downloaded at https://www.mdpi.com/article/10.3390/antibiotics15040393/s1; Table S1: Genome sequences retrieved from NCBI for pangenome analysis; Table S2: Antimicrobial resistance genes (identification and annotations) by sequence.

## Abbreviations

The following abbreviations are used in this manuscript:

CRAB	Carbapenem-resistant *Acinetobacter baumannii*
IC	International clones
MLST	Multi-Locus Sequence Typing

## References

[1] Gomes Chagas T.P., Rangel K., De-Simone S. (2024). Carbapenem-resistant Acinetobacter baumannii in Latin America. Acinetobacter baumannii-The Rise of a Resistant Pathogen.

[2] Li X., Li S., Zhou H., Hu L., Xia H., Xie L., Xie F. (2024). Nosocomial surveillance of multidrug-resistant Acinetobacter baumannii: A genomic epidemiological study. Microbiol. Spectr..

[3] AMarino A., Augello E., Stracquadanio S., Bellanca C.M., Cosentino F., Spampinato S., Cantarella G., Bernardini R., Stefani S., Cacopardo B. (2024). Unveiling the Secrets of Acinetobacter baumannii: Resistance, Current Treatments, and Future Innovations. Int. J. Mol. Sci..

[4] World Health Organization (2017). Guidelines for the Prevention and Control of Carbapenem-Resistant Enterobacteriaceae, Acinetobacter baumannii and Pseudomonas aeruginosa in Health Care Facilities.

[5] World Health Organization (2024). WHO Bacterial Priority Pathogens List, 2024.

[6] Sethuvel D.P.M., Bakthavatchalam Y.D., Karthik M., Irulappan M., Shrivastava R., Periasamy H., Veeraraghavan B. (2023). β-Lactam Resistance in ESKAPE Pathogens Mediated Through Modifications in Penicillin-Binding Proteins: An Overview. Infect. Dis. Ther..

[7] Thacharodi A., Vithlani A., Hassan S., Alqahtani A., Pugazhendhi A. (2024). Carbapenem-resistant Acinetobacter baumannii raises global alarm for new antibiotic regimens. iScience.

[8] Boutzoukas A., Doi Y. (2025). The global epidemiology of carbapenem-resistant Acinetobacter baumannii. JAC Antimicrob. Resist..

[9] Pearl S., Anbarasu A. (2025). Genomic landscape of nosocomial Acinetobacter baumannii: A comprehensive analysis of the resistome, virulome, and mobilome. Sci. Rep..

[10] Kubin C.J., Garzia C., Uhlemann A.C. (2025). Acinetobacter baumannii treatment strategies: A review of therapeutic challenges and considerations. Antimicrob. Agents Chemother..

[11] Hamidian M., Nigro S.J. (2019). Emergence, molecular mechanisms and global spread of carbapenem-resistant acinetobacter baumannii. Microb. Genom..

[12] Müller C., Reuter S., Wille J., Xanthopoulou K., Stefanik D., Grundmann H., Higgins P.G., Seifert H. (2023). A global view on carbapenem-resistant Acinetobacter baumannii. mBio.

[13] Rodríguez C.H., Nastro M., Famiglietti A. (2018). Carbapenemases in Acinetobacter baumannii. Review of their dissemination in Latin America. Rev. Argent. Microbiol..

[14] Shelenkov A., Mikhaylova Y., Akimkin V. (2024). Genomic Epidemiology Dataset for the Important Nosocomial Pathogenic Bacterium Acinetobacter baumannii. Data.

[15] Campos-Godínez J.F., Villegas-Campos M., Molina-Mora J.A. (2025). Core Perturbomes of Escherichia coli and Staphylococcus aureus Using a Machine Learning Approach. Pathogens.

[16] Molina Mora J.A., Montero-Manso P., García-Batán R., Campos-Sánchez R., Fernández J.V., García F. (2021). A first perturbome of Pseudomonas aeruginosa: Identification of core genes related to multiple perturbations by a machine learning approach. Biosystems.

[17] Ong S.W.X., Rao P., Khong W.X., Ong V.Y.F., Sridatta P.S.R., Thevasagayam N.M., Ho B.C.H., Ang B.S.P., De P.P., Ng O.T. (2023). Genomic surveillance uncovers ongoing transmission of carbapenem-resistant Acinetobacter baumannii (CRAB) and identifies actionable routes of transmissions in an endemic setting. Infect. Control Hosp. Epidemiol..

[18] Djordjevic S.P., Jarocki V.M., Seemann T., Cummins M.L., Watt A.E., Drigo B., Wyrsch E.R., Reid C.J., Donner E., Howden B.P. (2024). Genomic surveillance for antimicrobial resistance—A One Health perspective. Nat. Rev. Genet..

[19] Shelenkov A., Akimkin V., Mikhaylova Y. (2023). International Clones of High Risk of Acinetobacter Baumannii—Definitions, History, Properties and Perspectives. Microorganisms.

[20] Jolley K.A., Bray J.E., Maiden M.C.J. (2018). Open-access bacterial population genomics: BIGSdb software, the PubMLST.org website and their applications. Wellcome Open Res..

[21] Bartual S.G., Seifert H., Hippler C., Luzon M.A.D., Wisplinghoff H., Rodríguez-Valera F. (2005). Development of a multilocus sequence typing scheme for characterization of clinical isolates of Acinetobacter baumannii. J. Clin. Microbiol..

[22] Diancourt L., Passet V., Nemec A., Dijkshoorn L., Brisse S. (2010). The population structure of Acinetobacter baumannii: Expanding multiresistant clones from an ancestral susceptible genetic pool. PLoS ONE.

[23] Martinez I. Genomes Without Borders Bridging Gaps in Health Equity. https://www.neb.com/en/nebinspired-blog/genomes-without-borders-bridging-gaps-in-health-equity?srsltid=AfmBOopcB7Bqif-YJEL_EPNtIYYvdD3_TLTKUi6lbFzkVkc_3ZSdw4V7.

[24] World Health Organization (2025). Human Genomics Technologies in Clinical Studies—The Research landscape: Report on the 1990–2024 Period.

[25] Alvarez-Gomez R.M., De la Fuente-Hernandez M.A., Herrera-Montalvo L., Hidalgo-Miranda A. (2021). Challenges of diagnostic genomics in Latin America. Curr. Opin. Genet. Dev..

[26] Molina-Mora J.A., Reales-González J., Camacho E., Duarte-Martínez F., Tsukayama P., Soto-Garita C., Brenes H., Cordero-Laurent E., dos Santos A.R., Salgado C.G. (2023). Overview of the SARS-CoV-2 genotypes circulating in Latin America during 2021. Front. Public Health.

[27] Herrera Agüero S., Sosa A., Martinez A.A., Moreno A., Pereira C.R.C., Gonzalez C., Soto-Garita C., Ulate D., Cordero-Laurent E., Brenes H. (2026). Overview of SARS-CoV-2 genomic surveillance in Central America and the Dominican Republic from February 2020 to January 2023: The impact of PAHO and COMISCA’s collaborative efforts. Front. Public Health.

[28] Concha-Toloza M., González L.C., Estrella A.H.H., Porto D.F.D., Campos-Sánchez R., Molina-Mora J.A. (2024). Genomic, socio-environmental, and sequencing capability patterns in the surveillance of SARS-CoV-2 in Latin America and the Caribbean up to 2023. Sci. Rep..

[29] Radeino Ambe J., Bhan A., Silaigwana B., Salas S.P., Edwards S. (2026). Pathogen access and benefit sharing in a pandemic: Working towards fair exchange?. Lancet Microbe.

[30] Escandón-Vargas K., Reyes S., Gutiérrez S., Villegas M.V. (2017). The epidemiology of carbapenemases in Latin America and the Caribbean. Expert Rev. Anti. Infect. Ther..

[31] Kim U.J., Kim H.K., An J.H., Cho S.K., Park K.-H., Jang H.-C. (2014). Update on the Epidemiology, Treatment, and Outcomes of Carbapenem-resistant Acinetobacter infections. Chonnam Med. J..

[32] Ma C., McClean S. (2021). Mapping global prevalence of acinetobacter baumannii and recent vaccine development to tackle it. Vaccines.

[33] Gutiérrez K., Vásquez-Mendoza A., Rodríguez C. (2024). An outbreak of severe or lethal infections by a multidrug-resistant Acinetobacter baumannii ST126 strain carrying a plasmid with blaNDM-1 and blaOXA-58 carbapenemases. Diagn. Microbiol. Infect. Dis..

[34] Pillonetto M., Wink P.L., Melano R.G., Jiménez-Pearson M.A., Touchet N.L.M., Rojas S.Y.S., O Kulek D.N., Abreu A.L., Peral R.T., Miorando R. (2025). Carbapenemases producing gram-negative bacteria surveillance in Latin America and the caribbean: A retrospective observational study from 2015 to 2020. Lancet Reg. Health–Am.

[35] Castillo-Ramírez S., Aguilar-Vera A., Kumar A., Evans B. (2025). Acinetobacter baumannii: Much more than a human pathogen. Antimicrob. Agents Chemother..

[36] Gutierrez R., Wellcome Connecting Science (2025). Dissemination and evolution of large and megaplasmids linked to an NDM carbapenemase nationwide outbreak in Costa Rica. Applied Bioinformatics & Public Health Microbiology.

[37] Buitrago-Garcia D., Salanti G., Low N. (2022). Studies of prevalence: How a basic epidemiology concept has gained recognition in the COVID-19 pandemic. BMJ Open.

[38] Molina-Mora J.A., Rojas-Varela Á., Martínez-Arana C., Portilla-Victor L., Quirós-Fallas I., Sánchez-Fonseca M., Araya X., Cascante-Serrano D., Segura-Retana E., Espinoza-Solís C. (2025). Finding the Missing IMP Gene: Overcoming the Imipenemase IMP Gene Drop-Out in Automated Molecular Testing for Carbapenem-Resistant Bacteria Circulating in Latin America. Antibiotics.

[39] Liu H., Moran R.A., Doughty E.L., Hua X., Snaith A.E., Zhang L., Chen X., Guo F., van Schaik W., McNally A. (2024). Longitudinal genomics reveals carbapenem-resistant Acinetobacter baumannii population changes with emergence of highly resistant ST164 clone. Nat. Commun..

[40] Li S., Jiang G., Wang S., Wang M., Wu Y., Zhang J., Liu X., Zhong L., Zhou M., Xie S. (2025). Emergence and global spread of a dominant multidrug-resistant clade within Acinetobacter baumannii. Nat. Commun..

[41] Karah N., Faille N., Allard N., Grenier F., Abou-Fayad A., Higgins P.G., Al-Hassan L., Evans B.A., Poirel L., Bonnin R.A. (2025). Global emergence of Acinetobacter baumannii International Clone 12 predominantly found in the Middle East. Microb. Genom..

[42] Iovleva A., Fowler V.G., Doi Y. (2024). Treatment approaches for carbapenem-resistant Acinetobacter baumannii infections. Drugs.

[43] Bello-López E., Kawabata A., Cantero J., Mendoza S., Pertile E., Perez-Osegura A., Cevallos M.A., Peralta H., Aguilar-Vera A., Castillo-Ramirez S. (2025). Genomic epidemiology reveals antibiotic resistance transfer and polyclonal dissemination of Acinetobacter baumannii in a Paraguayan hospital. Antimicrob. Agents Chemother..

[44] Jeon W.J., Kim Y.J., Seo J.H., Yoo J.S., Moon D.C. (2024). Genomic Analysis of Carbapenem-Resistant Acinetobacter baumannii Isolated from Bloodstream Infections in South Korea. Antibiotics.

[45] Barrios Camacho H., Aguirre L.L., Duran Bedolla J. (2024). Genomic analysis of the main epidemiological lineages of Acinetobacter baumannii in Mexico. Front. Cell. Infect. Microbiol..

[46] Rafei R., Pailhoriès H., Hamze M., Eveillard M., Mallat H., Dabboussi F., Joly-Guillou M.-L., Kempf M. (2015). Molecular epidemiology of Acinetobacter baumannii in different hospitals in Tripoli, Lebanon using bla(OXA-51-like) sequence based typing. BMC Microbiol..

[47] Mindlin S., Maslova O., Beletsky A., Nurmukanova V., Zong Z., Mardanov A., Petrova M. (2021). Ubiquitous Conjugative Mega-Plasmids of Acinetobacter Species and Their Role in Horizontal Transfer of Multi-Drug Resistance. Front. Microbiol..

[48] Prity F.T., Tobin L.A., Maharajan R., Paulsen I.T., Cain A.K., Hamidian M. (2023). The evolutionary tale of eight novel plasmids in a colistin-resistant environmental Acinetobacter baumannii isolate. Microb. Genom..

[49] Hamidian M., Hawkey J., Wick R., Holt K.E., Hall R.M. (2019). Evolution of a clade of Acinetobacter baumannii global clone 1, lineage 1 via acquisition of carbapenem- and aminoglycoside-resistance genes and dispersion of ISAba1. Microb. Genom..

[50] Holt K., Kenyon J.J., Hamidian M., Schultz M.B., Pickard D.J., Dougan G., Hall R.M. (2016). Five decades of genome evolution in the globally distributed, extensively antibiotic-resistant Acinetobacter baumannii global clone 1. Microb. Genom..

[51] Álvarez V.E., Quiroga M.P., Galán A.V., Vilacoba E., Quiroga C., Ramírez M.S., Centrón D. (2020). Crucial Role of the Accessory Genome in the Evolutionary Trajectory of Acinetobacter baumannii Global Clone 1. Front. Microbiol..

[52] Imperi F., Antunes L.C., Blom J., Villa L., Iacono M., Visca P., Carattoli A. (2011). The genomics of Acinetobacter baumannii: Insights into genome plasticity, antimicrobial resistance and pathogenicity. IUBMB Life.

[53] Weber B.S., Ly P.M., Irwin J.N., Pukatzki S., Feldman M.F. (2015). A multidrug resistance plasmid contains the molecular switch for type VI secretion in Acinetobacter baumannii. Proc. Natl. Acad. Sci. USA.

[54] Wallace L., Daugherty S.C., Nagaraj S., Johnson J.K., Harris A.D., Rasko D.A. (2016). Use of Comparative Genomics To Characterize the Diversity of Acinetobacter baumannii Surveillance Isolates in a Health Care Institution. Antimicrob. Agents Chemother..

[55] Mateo-Estrada V., Fernández-Vázquez J.L., Moreno-Manjón J., Hernández-González I.L., Rodríguez-Noriega E., Morfín-Otero R., Alcántar-Curiel M.D., Castillo-Ramírez S. (2021). Accessory Genomic Epidemiology of Cocirculating Acinetobacter baumannii Clones. mSystems.

[56] Decano A.G., Ludden C., Feltwell T., Judge K., Parkhill J., Downing T. (2019). Complete Assembly of Escherichia coli Sequence Type 131 Genomes Using Long Reads Demonstrates Antibiotic Resistance Gene Variation within Diverse Plasmid and Chromosomal Contexts. mSphere.

[57] Zhao W., Zeng W., Pang B., Luo M., Peng Y., Xu J., Kan B., Li Z., Lu X. (2023). Oxford nanopore long-read sequencing enables the generation of complete bacterial and plasmid genomes without short-read sequencing. Front. Microbiol..

[58] Johnson J., Soehnlen M., Blankenship H.M. (2023). Long read genome assemblers struggle with small plasmids. Microb. Genom..

[59] Massik A., Hibaoui L., Benboubker M., Yahyaoui G., Oumokhtar B., Mahmoud M. (2023). Acinetobacter baumannii Carbapenemase Producers in Morocco: Genetic Diversity. Cureus.

[60] Molina-Mora J.-A., Campos-Sánchez R., Rodríguez C., Shi L., García F. (2020). High quality 3C de novo assembly and annotation of a multidrug resistant ST-111 Pseudomonas aeruginosa genome: Benchmark of hybrid and non-hybrid assemblers. Sci. Rep..

[61] Rojas-Miranda H., Madrigal-Ly V., Molina-Mora J.A. (2025). Benchmarking genome assemblers for four bacterial models based on contiguity, correctness, and completeness. Sci. Rep..

[62] Chen Z., Erickson D.L., Meng J. (2020). Benchmarking hybrid assembly approaches for genomic analyses of bacterial pathogens using Illumina and Oxford Nanopore sequencing. BMC Genom..

[63] Molina-Mora J.A., Chinchilla-Montero D., García-Batán R., García F. (2021). Genomic context of the two integrons of ST-111 Pseudomonas aeruginosa AG1: A VIM-2-carrying old-acquaintance and a novel IMP-18-carrying integron. Infect. Genet. Evol..

[64] Hajikhani B., Sameni F., Ghazanfari K., Abdolali B., Yazdanparast A., Dezfuli A.A., Nasiri M.J., Goudarzi M., Dadashi M. (2023). Prevalence of blaNDM-producing Acinetobacter baumannii strains isolated from clinical samples around the world; a systematic review. Gene Rep..

[65] Mangas E.L., Rubio A., Álvarez-Marín R., Labrador-Herrera G., Pachón J., Pachón-Ibáñez M.E., Divina F., Pérez-Pulido A.J. (2019). Pangenome of Acinetobacter baumannii uncovers two groups of genomes, one of them with genes involved in CRISPR/Cas defence systems associated with the absence of plasmids and exclusive genes for biofilm formation. Microb. Genom..

[66] Bhat T., Kumar M., Ballamoole K.K., Deekshit V.K., Gollapalli P. (2025). Pangenome-based network analysis of Acinetobacter baumannii reveals the landscape of conserved therapeutic targets. Mol. Divers.

[67] Hassan A., Naz A., Obaid A., Paracha R.Z., Naz K., Awan F.M., Muhmmad S.A., Janjua H.A., Ahmad J., Ali A. (2016). Pangenome and immuno-proteomics analysis of Acinetobacter baumannii strains revealed the core peptide vaccine targets. BMC Genom..

[68] Strobeyko A. (2024). The Future of Pathogen Access and Benefit Sharing under International Law. Verfassungsblog.

[69] Andrews S. FastQC A Quality Control Tool for High Throughput Sequence Data. https://www.bioinformatics.babraham.ac.uk/projects/fastqc/.

[70] Bolger A.M., Lohse M., Usadel B. (2014). Trimmomatic: A flexible trimmer for Illumina sequence data. Bioinformatics.

[71] Li D., Liu C.-M., Luo R., Sadakane K., Lam T.-W. (2015). MEGAHIT: An ultra-fast single-node solution for large and complex metagenomics assembly via succinct de Bruijn graph. Bioinformatics.

[72] Wick R.R., Judd L.M., Gorrie C.L., Holt K.E. (2017). Unicycler: Resolving bacterial genome assemblies from short and long sequencing reads. PLoS Comput. Biol..

[73] Molina-Mora J.A., Garcia F.G. (2020). The 3C criterion: Contiguity, Completeness and Correctness to assess de novo genome assemblies. BMC Bioinform. Bioinform. Algorithms Appl..

[74] Seemann T. (2014). Prokka: Rapid prokaryotic genome annotation. Bioinformatics.

[75] Arndt D., Grant J.R., Marcu A., Sajed T., Pon A., Liang Y., Wishart D.S. (2016). PHASTER: A better, faster faster version of the PHAST phage search tool. Nucleic Acids Res..

[76] Langille M.G.I., Brinkman F.S.L. (2009). IslandViewer: An integrated interface for computational identification and visualization of genomic islands. Bioinformatics.

[77] Robertson J., Nash J.H.E. (2018). MOB-suite: Software tools for clustering, reconstruction and typing of plasmids from draft assemblies. Microb. Genom..

[78] Page A.J., Cummins C.A., Hunt M., Wong V.K., Reuter S., Holden M.T.G., Fookes M., Falush D., Keane J.A., Parkhill J. (2015). Roary: Rapid large-scale prokaryote pan genome analysis. Bioinformatics.

[79] Letunic I., Bork P. (2019). Interactive Tree Of Life (iTOL) v4: Recent updates and new developments. Nucleic Acids Res..

